# Preliminary Report on the Pathological Findings in the Nerves Following War Injuries

**Published:** 1918

**Authors:** 


***Preliminary Report on the Pathological Findings in the Nerves Following War Injuries.*** By Captain SYDNEY M. CONE, M. R. C. Abs. from the *Brit. Med. Jour.*, Nov. 10, 1917.

The author insists that without a good stain for axis cylinder and medullary sheath one cannot do nerve work in the laboratory. His technique is a follows :

Specimens are hardened in a 10 per cent formalin solution and then carried through the alcohols to ether and absolute alcohol and celloidin. Thin sections are preferable, but good results may be obtained with section 20 m thick.

The section is washed in water and placed in carbol-fuchsin fifteen minutes, momentarily in water, 1 per cent osmic acid 5 minutes, water 2 minutes, acid alcohol 1 per cent 10 seconds, 95 per cent. alcohol 5 minutes. Absolute alcohol and oil of cloves are now used alternately until the section appears deep pink but translucent; it is then placed in xylol for two minutes and mounted in Canada balsam.

The author notes particulary the irregularity of growth of new safciculi of nerves in the proximal end of the nerve about the old parallel running fibers as far as the bulbous enlargement at the wound.

The operation aims to expose the nerve, — with due care regarding all anatomical relations, — and the incisions are made in a line parallel to the fibers. Excisions of the nerve are then made leaving greyish-pink, moderately soft nerve-ends. Suture is performed with chromicized catgut. Cargile membrane, fascia, or muscle covering are used to protect the area of suture from future adhesions.

The author adds a series of observations regarding nerve growth :

In some cases no fibers at all were to be seen at the bulbous enlargement but such was only rarely the case.

Wherever young fibrils were found, there was great cell proliferation.

The adhesion invariably contained cellular young nerves, some with a faint line of myelin covering the axis cylinders. Others were well protected by a medullary sheath and sheath of Schwann. A great number of neuroblasts were observed in the adhesions particularly at the distal end.

The author mentions especially the cones (barbs or "*renflements biconiques*" in the youngest fibrils of adults or embryo, and also the vacuoles ("*gonflements*") of myelin moniliform appearance of fibrils, due to the neurokeratin framework.

